# The adaptability of *Pseudomonas aeruginosa* biofilm in oxygen-limited environments

**DOI:** 10.3389/fcimb.2025.1655335

**Published:** 2025-09-19

**Authors:** Ling Ren, Yang Yuan, Khaled Farea, Xu Feng, Jia He, Yi Liu, Bowen Zheng

**Affiliations:** ^1^ Department of Orthodontics, School and Hospital of Stomatology, China Medical University, Shenyang, Liaoning, China; ^2^ Shenyang Clinical Medical Research Center of Orthodontic Disease, Shenyang, China

**Keywords:** biofilms, oxygen-limited, anaerobic metabolism, transcriptional factors, two-component systems, quorum sensing, *Pseudomonas aeruginosa*

## Abstract

Under oxygen-limited conditions, the adaptability and underlying mechanisms of bacterial biofilms have become key areas of interest in microbiology and clinical infection research. Within biofilms—composed of bacterial communities and extracellular matrix—an oxygen gradient commonly forms, resulting in hypoxic or even anoxic microenvironments. Such conditions substantially increase biofilm antibiotic resistance and facilitate the persistence of chronic infections. This review systematically summarizes the adaptive strategies employed by biofilms in hypoxic environments, including anaerobic metabolism, phenazine-mediated electron shuttling, and virulence factor regulation. These adaptive responses are governed by genes involved in anaerobic metabolism, quorum sensing systems, and the secondary messenger 3,5-cyclic diguanylic acid (c-di-GMP), which collectively influence biofilm formation. Key transcriptional regulators such as Anr and Dnr, the two-component system NarXL, along with specific functional genes, form an intricate regulatory network. This article aims to provide a comprehensive overview of the adaptive mechanisms of Pseudomonas aeruginosa biofilms under oxygen-limited conditions, providing a theoretical foundation for the development of novel anti-infective therapies, targeting the biofilm infection microenvironment in cystic fibrosis and chronic wounds.

## 1 Introduction

*Pseudomonas aeruginosa* is a widely distributed Gram-negative bacterium known to cause nosocomial infections and potentially fatal infections in immunocompromised patients (Gale et al., 2015; Gomila et al., 2018). It is also a persistent colonizer of the lungs in cystic fibrosis (CF) patients, where it is notoriously difficult to eradicate (Singh et al., 2000). During chronic infections, *P. aeruginosa* predominantly exists in biofilm form. Bacterial biofilms adhere to biological or abiotic surfaces and consist of bacterial communities embedded within an extracellular matrix (ECM), comprising proteins, polysaccharides, and extracellular DNA (eDNA), among other components (Vestby et al., 2020). These molecules provide structural integrity and facilitate intercellular adhesion (Schilcher and Horswill, 2020; Chiba et al., 2022). Unlike planktonic bacteria, biofilms exhibit altered growth rates, metabolism, and gene expression profiles (Donlan, 2002). Bacteria in the inner layers of biofilms adapt to low metabolic activity and undergo anaerobic respiration. Due to the protective barrier of the ECM, biofilm-associated bacteria demonstrate increased antibiotic resistance by approximately 10- to 1000-fold (Wagner and Iglewski, 2008; Stokes et al., 2019). Consequently, biofilms are inherently difficult to treat, leading to persistent and chronic infections that pose significant clinical challenges (Zhao et al., 2023a). It is estimated that 65% to 80% of human bacterial infections are associated with biofilms (Zhao et al., 2023a; Römling and Balsalobre, 2012), underscoring the critical need to eradicate pathogenic biofilms for effective management of chronic infections.

Biofilm formation is a complex process influenced by various external factors, including nutrients, osmotic pressure, temperature, and oxygen availability (Lin et al., 2012). The human body presents numerous low-oxygen or anaerobic niches. For example, *P. aeruginosa* grows as biofilms in the lungs of CF patients, where chronic infection leads to hypoxic or even anaerobic conditions (Martin et al., 2023; Hall-Stoodley and McCoy, 2022). Similarly, *Salmonella* colonizes anaerobic niches within the intestinal tract (Ehrhardt et al., 2023), and oxygen concentrations in infected or necrotic tissues and wounds are notably low (Carreau et al., 2011). *In vitro* studies have confirmed that oxygen gradients are commonly present in bacterial biofilms, including those formed by *P. aeruginosa* (Papa et al., 2023), *Staphylococcus aureus* (Zamudio-Chávez et al., 2023), and *Enterococcus faecalis* (James et al., 2016), particularly in mature biofilms (Borriello et al., 2004). This oxygen limitation enhances pathogen virulence and survival (Wang et al., 2022). Using oxygen microelectrode technology, localized hypoxia within *P. aeruginosa* biofilms has been confirmed, which restricts protein synthesis in mature biofilm interiors and contributes to antibiotic resistance (Høiby et al., 2024). Oxygen deficiency accounts for at least 70% of antibiotic resistance in mature *P. aeruginosa* biofilm cells, highlighting oxygen concentration as a critical factor in biofilm formation and multidrug resistance stability. Understanding how bacteria rapidly sense and respond to oxygen-limited environments is essential for improving infection treatments. This review summarizes the adaptations and mechanisms of *P. aeruginosa* biofilms under oxygen-limited conditions, aiming to provide a novel approach for eradicating biofilms through modulation of the infection microenvironment.

## 2 Formation of oxygen-limited microenvironment

**Figure 1:**
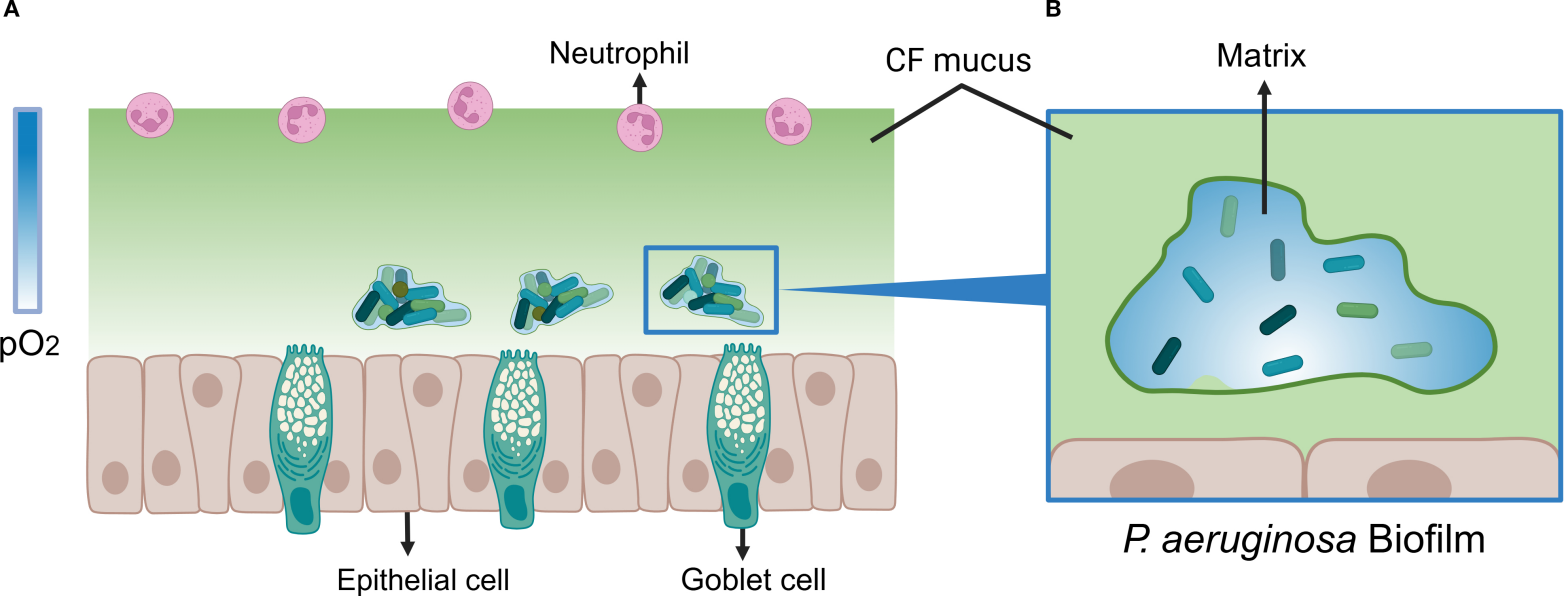
Model of *P. aeruginosa* Biofilm Under Oxygen-Limited Conditions. **(A)** *P. aeruginosa* biofilm model in cystic fibrosis (CF) mucus under oxygen-limited conditions. The color bar on the left side of the figure represents the partial pressure of oxygen (pO_2_). The gradient from deep to light blue indicates an increasing anaerobic state within the mucus. Goblet cells continuously secrete mucus, and combined with elevated oxygen consumption by host cells, a steep oxygen gradient forms within the thickened CF mucus (depicted in light green). *P. aeruginosa* forms biofilms within the hypoxic regions of the mucus, provoking a chronic inflammatory response in the host. This response leads to neutrophil infiltration and a respiratory burst, which further depletes oxygen levels within the mucus. **(B)** Oxygen-Limited Microenvironment within *P. aeruginosa* Biofilm. The transition from deep to light blue illustrates the gradual decrease in oxygen concentration observed inside the biofilm. Oxygen depletion occurs predominantly in the deep regions (white area). This gradient arises because oxygen is directly consumed by host cells and bacterial respiration at the biofilm surface, leading to a progressive oxygen decline with increasing distance from the surface. Created with BioRender.com.

The oxygen concentration gradient is an important characteristic of bacterial biofilms. The oxygen concentration in the environment is mostly around 19.95%. Generally, an oxygen concentration ranging from 11% to 1% is regarded as hypoxic (Carreau et al., 2011), while an environment with less than 1% oxygen or completely devoid of oxygen is an anoxic environment (Mashruwala et al., 2017). In CF lungs, *P. aeruginosa* forms biofilms where oxygen penetration is severely restricted. Microelectrode measurements reveal that oxygen levels decline progressively with depth and penetrate only 50 μm from the biofilm surface (Werner et al., 2004), whereas the average biofilm thickness can reach 210 μm (Borriello et al., 2004). Consequently, cells deep within the biofilm experience hypoxic or anoxic conditions (Werner et al., 2004; Dietrich et al., 2013). Active protein synthesis is confined to a zone roughly 30 μm above the base (Xu et al., 1998). This oxygen limitation arises from both physical and biological factors: the dense biofilm matrix and viscous CF mucus impede diffusion, while host inflammatory responses recruit neutrophils whose respiratory burst consumes oxygen (Kolpen et al., 2010). Additionally, oxygen is consumed directly by host and bacteria respiration at the biofilm periphery, maintaining a persistent hypoxic gradient (Kolpen et al., 2010), as shown in **Figure 1**. Sputum from CF patients is predominantly micro-aerobic to anaerobic (Yoon et al., 2002; Alvarez-Ortega and Harwood, 2007a; Hassett et al., 2009). The extent of anoxic regions correlates with bacterial load and mucus thickness, potentially occupying large portions of mucus volume (Cowley et al., 2015). Notably, multidrug resistance protein (MexA) is more abundant in anoxic zones, suggesting enhanced drug tolerance under hypoxia in *P. aeruginosa* (Schaible et al., 2012; Martin et al., 2023; Pulukkody et al., 2021). Despite being a facultative anaerobe, *P. aeruginosa* can maintain growth under oxygen-limited conditions (Jackson et al., 2014), which in turn promotes robust biofilm formation (Rossi et al., 2021).

## 3 The effect of oxygen-limited conditions on *P. aeruginosa* biofilms

### 3.1 Anaerobic metabolism

**Figure 2:**
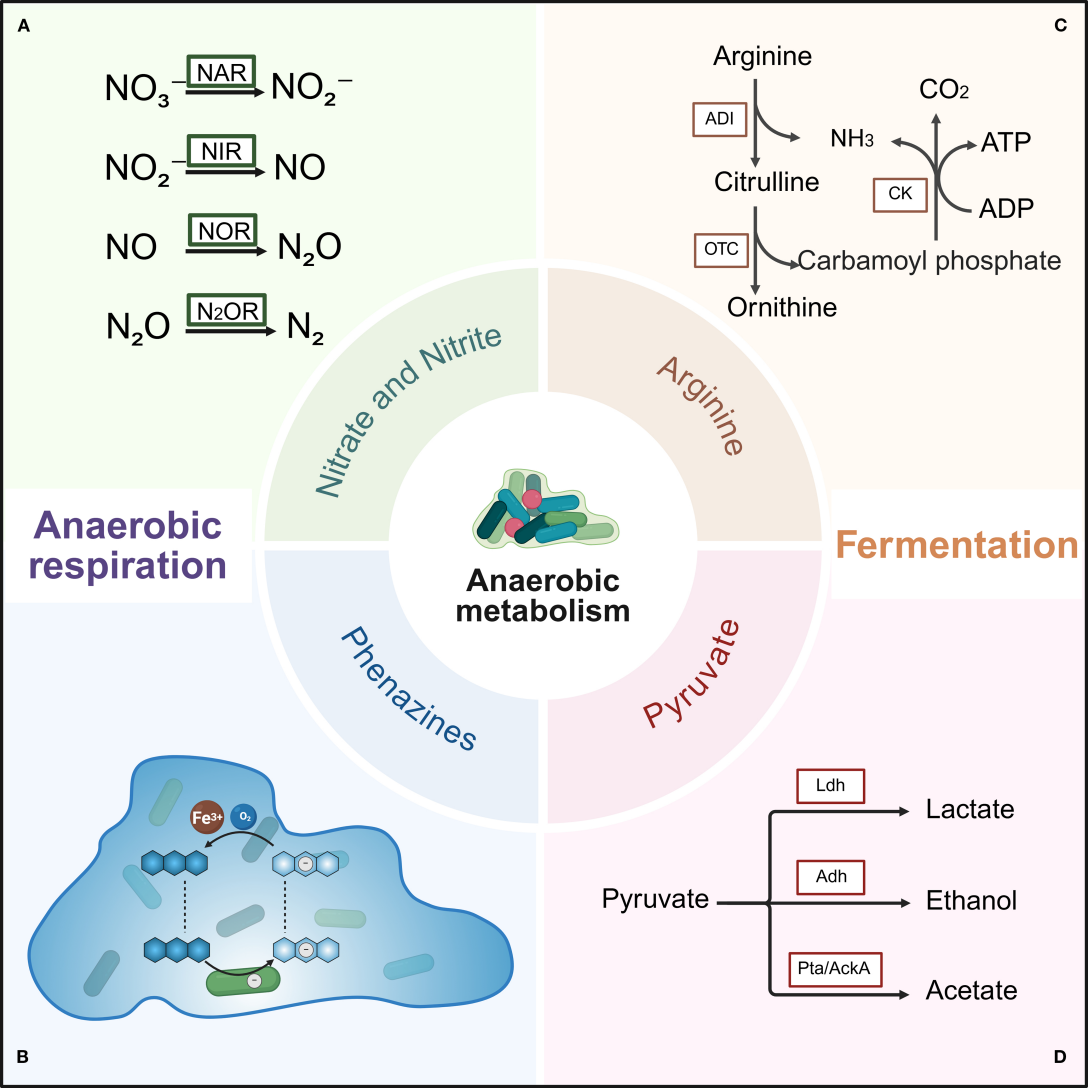
Anaerobic metabolism of *P. aeruginosa* biofilms under oxygen-limited conditions. Two primary anaerobic metabolic pathways are shown: anaerobic respiration and fermentation. Anaerobic respiration mainly involves denitrification pathways utilizing nitrate and phenazine to maintain cellular redox balance. Fermentation includes the arginine and pyruvate fermentation pathways, which generate ATP to sustain bacterial survival. **(A)** Reduction of nitrate, nitrite, nitric oxide, and nitrous oxide by nitrate reductase, nitrite reductase, nitric oxide reductase, and nitrous oxide reductase, respectively. **(B)** Electron transfer cycle of phenazine within biofilm. Cells are represented as rods, phenazine as blue hexagons, electrons as white circles, and oxygen concentration is depicted by the blue background. **(C)** Arginine deiminase (ADI) pathway. Key enzymes include arginine deiminase (ADI), ornithine carbamoyltransferase (OTC), and carbamate kinase (CK). **(D)** Pyruvate fermentation pathway. Key enzymes include lactate dehydrogenase (Ldh), alcohol dehydrogenase (Adh), phosphate acetyltransferase (Pta), and acetate kinase (AckA). Created with BioRender.com.

In human host environments, pathogens encounter fluctuating oxygen levels, with adaptive responses to such fluctuations primarily occurring at the metabolic level (Ferreira et al., 2013). Metabolism reprogramming is central to initiating bacterial tolerance mechanisms and reactivating transitions from a non-replicating to an actively growing state. *P. aeruginosa* exhibits remarkable metabolic versatility, utilizing diverse catabolic and anabolic pathways encoded in its genome to thrive in harsh environments (La Rosa et al., 2018; Crabbé et al., 2019). While aerobic metabolism dominates under oxygen-replete conditions, biofilm-embedded bacteria frequently reside in oxygen-limited niches. Under such constraints, *P. aeruginosa* shifts to aerobic metabolism—enabling sustained growth and metabolic activity without oxygen (Yoon et al., 2002). This adaptation encompasses anaerobic respiration and fermentation, as illustrated in **Figure 2**. Critically, this metabolic switch confers enhanced antibiotic tolerance and specific molecule resistance (Peng et al., 2017).

For energy generation via respiratory, *P. aeruginosa* can utilize oxygen, nitrogen compounds, and potentially other electron acceptors such as thiosulfate (Rossi et al., 2021). Under oxygen-limited conditions, *P. aeruginosa* performs anaerobic respiration using nitrate, nitrite, or nitrous oxide as terminal electron acceptors, facilitating rapid growth and energy production. In the absence of nitrate and nitrite, *P. aeruginosa* employs two alternative fermentation pathways to support slow growth or survival. The first involves substrate-level phosphorylation through arginine utilization, resulting in very slow growth. The second fermentation pathway uses pyruvate as a substrate, converting it into acetate, lactate, and small amounts of succinate.

#### 3.1.1 Anaerobic respiration

Nitrogen metabolism under anaerobic and biofilm conditions promotes the virulence and tolerance of pathogens under hypoxic stress. Denitrification, a continuous four-step, eight-electron reduction process converting nitrate to nitrogen, is a key pathway enabling *P. aeruginosa* to respire anaerobically under oxygen-limited conditions (Yoon et al., 2002; Stoodley et al., 2024). In this process, nitrate (NO_3_^-^) or nitrite (NO_2_^-^) serves as the terminal electron acceptor (TEA), replacing oxygen generate energy. Denitrification also serves as an important cellular redox balancing mechanism within biofilms, provided these electron acceptors are available in sufficient concentrations (Line et al., 2014; Palmer et al., 2007). Using an alginate-encapsulated *P. aeruginosa* chronic infection model, it has been demonstrated that oxygen depletion limits bacterial growth. Supplementing nitrate as an alternative electron acceptor sustains the growth of *P. aeruginosa* microcolonies under oxygen-limited conditions, although the overall respiration rate decreases (Sønderholm et al., 2017). Consistent with these findings, lower nitrate and nitrite levels have been observed in infected wounds compared to non-infected controls, reflecting bacterial consumption via denitrification (Debats et al., 2006). Clinically, the ability of *P. aeruginosa* to perform anaerobic respiration under hypoxic conditions contributes to enhanced biofilm formation (Balasubramanian et al., 2017), aligning with model predictions (Aristotelous, 2022).

During hypoxic growth, nitric oxide (NO) can be produced endogenously as a denitrification intermediate or derived from exogenous NO donors. NO exhibits potent bactericidal properties (Fang, 1997). Accumulation of NO under anaerobic conditions acts as a stress signal, ultimately promoting biofilm formation as a defense mechanism (Yoon et al., 2011; Cutruzzolà and Frankenberg-Dinkel, 2016). NO detoxification proteins, including NO reductase (NOR, encoded by *norVW*) and flavohemoglobin (Hmp), help mitigate NO toxicity during denitrification (Gardner et al., 2003). Interestingly, nitrite reductase (NIR) also has NO-independent functions; NO can indirectly induce NIR expression and regulate flagella biosynthesis and swimming motility by forming a ternary complex with the molecular chaperone DnaK and flagellin FliC in the periplasm, serving as a scaffold (Borrero-de Acuña et al., 2015).

Beyond anaerobic respiration, *P. aeruginosa* in the mucus layer of CF lungs can perform microaerobic respiration. This process rapidly consumes oxygen, creating an oxygen gradient (Alvarez-Ortega and Harwood, 2007b). In the CF lung microenvironment, microaerobic respiration can occur simultaneously with nitrate respiration when both oxygen and nitrate are present (Chen et al., 2006). Three high-affinity terminal oxidases—the *cbb_3_-1*, *cbb_3_-2*, and cyanide-insensitive oxidases—enable growth at low oxygen concentrations and facilitate microaerobic growth. Notably, the genes encoding *cbb_3–_2* oxidase and the cyanide-insensitive oxidase (*cioAB)* are highly expressed under oxygen-limited conditions (Alvarez-Ortega and Harwood, 2007b). The cyanide-insensitive oxidases not only increase under microaerobic conditions but may also protect cells against hydrogen cyanide toxicity during growth. The *cbb_3–_1* oxidase is consistently expressed across various oxygen levels, suggesting *P. aeruginosa* maintains preparedness for sudden oxygen depletion without needing to trigger a transcriptionally regulated hypoxic response (Alvarez-Ortega and Harwood, 2007b).

#### 3.1.2 Fermentation

Under oxygen-limited and nitrate-depleted conditions, *P. aeruginosa* biofilms sustain other energy supply through fermentation, primarily by activating the arginine fermentation pathway (Scribani Rossi et al., 2022) and pyruvate fermentation (Eschbach et al., 2004), which moderately support anaerobic growth and survival. L-arginine serves as a substrate for ATP production, enabling bacterial persistence under these conditions (Scribani Rossi et al., 2022). Specifically, *P. aeruginosa* utilizes the arginine deiminase (ADI) pathway to generate energy under oxygen-limited conditions, producing 1 mole of ATP per mole of L-arginine consumed. When arginine concentrations are sufficiently high, substrate-level phosphorylation can yield enough ATP to maintain bacterial growth. Thus, denitrification and arginine fermentation represent core metabolic processes in *P. aeruginosa* under oxygen-limited conditions.

Additionally, under anaerobic conditions, *P. aeruginosa* ferments pyruvate into lactate, acetate, and succinate. Although pyruvate fermentation does not support substantial anaerobic growth, it promotes long-term bacterial viability without contributing directly to proliferation (Eschbach et al., 2004). Proteomic analyses of hypoxic biofilm regions further suggest that cells produce fermentation by-products such as acetate (Pulukkody et al., 2021). Notably, nitrate respiration inhibits pyruvate fermentation, whereas arginine fermentation proceeds independently of pyruvate metabolism (Eschbach et al., 2004).

### 3.2 Phenazines as an electron shuttle

*P. aeruginosa* is well known for producing colored, redox-active metabolites called phenazines (Glasser et al., 2017). These phenazine compounds produced vary in structure and chemical properties (Mavrodi et al., 2010), functioning as electron-cycling molecules. The redox cycling of phenazine involves alternating reduction and oxidation reactions, which reoxidize accumulated NADH, thereby facilitating the transfer of electrons from reducing agents such as NAD(P)H to oxidizing agents like oxygen. This process promotes adenosine triphosphate (ATP) production and the generation of proton-motive force, allowing cells to survive in hypoxic regions and supporting colony growth (Dietrich et al., 2013; Ciemniecki and Newman, 2020). The redox potential of phenazines enables their reduction by bacterial cells and subsequent reaction with higher-potential oxidizing agents outside the cells, such as ferric iron and oxygen (Glasser et al., 2014). Acting as electron shuttles between bacteria and external substrates (Wang and Newman, 2008), phenazines alleviate limitations posed by scarce electron acceptors (Dietrich et al., 2013). Effectively, within the deep layers of the biofilm community, electrons accumulated for ATP synthesis can be accepted by oxidized phenazines and transferred to extracellular oxidants like oxygen. The *cbb_3_*-type terminal oxidases Cco1 and Cco2 of *P. aeruginosa*, key components of the respiratory chain, participate in phenazine reduction (Jo et al., 2017).

Phenazines and the cellular redox state directly influence biofilm morphogenesis via the regulatory protein RmcA, which modulates matrix components responsible for wrinkle formation (Dietrich et al., 2008; Okegbe et al., 2017). Phenazine-producing colonies tend to grow smoothly, whereas phenazine-deficient strains exhibit rougher, highly wrinkled biofilms that maximize oxygen contact (Kempes et al., 2014). Wrinkling serves as an adaptive mechanism to optimize oxygen accessibility and maintain metabolic homeostasis. Beyond their redox roles, phenazines act as signaling molecules that promote biofilm formation. *P. aeruginosa* synthesizes phenazine pigments such as pyocyanin, which intercalate into DNA base-pair regions, enhancing electron transfer, causing structural perturbations, and increasing DNA viscosity. The interaction is crucial for biofilm development (Das et al., 2015). Disrupting the pyocyanin—DNA interaction—via antioxidants or other inhibitors—can impede biofilm formation and associated infections (Das et al., 2015). The redox cycling of pyocyanin also generates reactive oxygen species (ROS), which damage host cells and pathogen cells, releasing eDNA (Das and Manefield, 2012; Rada et al., 2013). Phenazine compounds also mediate efficient extracellular electron transfer (EET) by interacting with eDNA in *P. aeruginosa* biofilms (Saunders et al., 2020). Together with the anaerobic stress responses, phenazines promote antibiotic tolerance and contribute to disease progression (Schiessl et al., 2019; Jiménez Otero et al., 2023). Notably, phenazines enhance biofilm tolerance to antibiotics such as ciprofloxacin (Schiessl et al., 2019).

Phenazines also stimulate pyruvate fermentation under anoxic conditions by mediating expression of the *ackA* and *pta* genes required for this pathway (Glasser et al., 2014). Through their redox cycling, phenazines enable *P. aeruginosa* to oxidize pyruvate to acetate and couple acetate metabolism with ATP synthesis via acetate kinase, thereby enhancing survival. In pyruvate fermentation, ATP generation is tightly linked to redox balance, a contrast to the arginine fermentation pathway where this connection is absent (Glasser et al., 2014).

### 3.3 Virulence expression

Mathematical modeling studies have demonstrated that bacterial biofilms growing anaerobically in anoxic environments secrete elevated levels of toxins. These toxins diffuse through the environment and lyse neutrophils, helping the biofilm resist neutrophil-mediated attacks. Consequently, bacterial adaptability and biofilm formation are enhanced under these conditions (Aristotelous, 2022). *P. aeruginosa* biofilms express a highly regulated protein secretion apparatus known as the type III secretion system (T3SS), which planktonic cells cannot deploy (Mikkelsen et al., 2009). The T3SS directly translocates a specific subset of exotoxin effector proteins–including ExoS, ExoU, ExoT, and ExoY–into host cells, driving *P. aeruginosa* pathogenicity (Cowell et al., 2005; Taylor and Winter, 2020). Multiple studies indicate that bacteria mount adaptive responses to diverse environments by modulating gene expression and protein production, thereby regulating the expression of virulence factors (Cullen and McClean, 2015; Teng et al., 2018) and inducing virulence protein synthesis (Fang et al., 2016). Oxygen limitation is a critical regulator of T3SS expression, a major virulence determinant in *P. aeruginosa*. Exposure to hypoxic conditions activates the T3SS (O’Callaghan et al., 2011; Chung et al., 2013). This activation strongly depends on the glyoxylate shunt enzyme isocitrate lyase (ICL, encoded by *aceA*), which is highly expressed in cystic fibrosis isolates specifically under oxygen-limited conditions (Chung et al., 2013). ICL-dependent regulation influences the expression of the T3 structural proteins, effectors, and regulatory proteins (ExsC, ExsD, and ExsE). Additionally, *aceA* modulates biofilm formation by affecting the expression of *pslA*, a gene involved in the biosynthesis of an extracellular polysaccharide (Chung et al., 2013). Notably, *aceA* mutants display enhanced biofilm formation during anaerobic growth.

The RetS/LadS signaling pathway reciprocally regulates T3SS expression and biofilm formation through a complex mechanism involving the GacS/GacA two-component system, the small regulatory RNAs RsmZ and RsmY, and the translational repressor RsmA (Goodman et al., 2004; Goodman et al., 2009; Kay et al., 2006; Ventre et al., 2006; O’Callaghan et al., 2011). Activation of LadS—or downregulation of RetS—promotes GacS homodimer formation, resulting in GacA phosphorylation and activation, accompanied by increased production of *rsmZ* and *rsmY*. These small RNAs sequester RsmA, relieving its repression and thereby activating biofilm formation (Kay et al., 2006; Brencic et al., 2009). Conversely, RetS activation induces heterodimer formation with GacS, inhibiting the GacS/GacA pathway. Free RsmA then binds specific mRNA targets, modulating their stability and indirectly activating *exsA*-dependent T3SS expression (Brencic et al., 2009). The regulation of AceA may be mediated by the RetS/LadS signaling pathway (Chung et al., 2013).

Moreover, the anaerobic regulator Anr senses oxygen limitation and induces expression of *narL* within the NarL/NarX two-component system. NarL represses the RsmA-antagonistic RNAs *rsmZ* and *rsmY*, resulting in increased levels of free RsmA, which stimulates T3SS expression. Free RsmA positively regulates T3SS and possibly other virulence determinants under its control, serving as a convergence point for *P. aeruginosa*’s response to various environmental cues (O’Callaghan et al., 2011).

Finally, *P. aeruginosa* OprG, an outer membrane protein belonging to the OmpW family of eight β-barrel porins, is broadly distributed. In iron-rich anaerobic environments, ANR significantly upregulates oprG transcription. Purified OprG forms cation-selective channels and substantially enhances cytotoxicity (McPhee et al., 2009).

## 4 Regulatory mechanisms under oxygen-limited conditions

*P. aeruginosa* biofilms adapt to oxygen-limited conditions through coordinated regulatory mechanisms. Anaerobic metabolism, including denitrification, arginine fermentation, and pyruvate fermentation, is activated under oxygen-limited conditions. Additionally, quorum sensing systems and the secondary messenger cyclic di-GMP (c-di-GMP) play crucial roles in modulating biofilm formation in response. Transcriptional regulatory networks involving the transcription factors ANR and DNR, and the NarXL two-component system, orchestrate the biofilm’s adaptive responses under oxygen-limited environments. Moreover, recent studies have also identified specific genes that further support biofilms growth and development under such conditions.

### 4.1 Anaerobic metabolism-related genes

#### 4.1.1 Denitrification regulatory genes

The expression of *narI* and *nirS* genes in *P. aeruginosa* is upregulated under anaerobic conditions, playing key roles in anaerobic respiration (Martin et al., 2023). Notably, *P. aeruginosa* may induce denitrification genes in response to low oxygen levels regardless of nitrate availability. During anaerobic growth, two nitrate reductase gene clusters are expressed, one encoding a membrane-bound enzyme complex (*narGHJI*) associated with the cytoplasmic membrane, and another encoding a periplasmic enzyme complex (*napAB*) (Palmer et al., 2007). The membrane-bound nitrate reductase is essential for anaerobic growth, as *P. aeruginosa* depends on it for energy generation when nitrate is present (Palmer et al., 2007). Enzymes involved in nitrate respiration, including nitrate reductases NapA and NarG, accumulate in biofilms (Palmer et al., 2007). Furthermore, antibodies against NapA and NarG have been detected in the serum of CF patients, confirming *in vivo* production of these respiratory enzymes by (Palmer et al., 2007). The periplasmic nitrate reductase is not essential for anaerobic energy generation. However, it may balance intracellular redox states under low oxygen, a role reported in other microorganisms (Richardson et al., 2001). It is plausible that *P. aeruginosa* utilizes the periplasmic nitrate reductase similarly. The Nar complex, located in the cytoplasmic membrane, drives proton-motive force generation and ATP synthesis, whereas the periplasmic Nap complex mainly balances intracellular redox states without contributing directly to the transmembrane electrochemical gradient (Richardson et al., 2001; Williams et al., 2007; Borrero-de Acuña et al., 2016). This view contrasts with earlier findings where the periplasmic nitrate reductase and nitrate transport genes (e.g., *narK2*) were downregulated during anaerobic nitrate growth, with no observed differential regulation of the membrane-bound reductase (Filiatrault et al., 2005; Sharma et al., 2006). The discrepancy may stem from methodological differences, as the prior study lacked saturating mutagenesis and thus might have missed mutants in the *nar* operon. The *narG* gene is critical for anaerobic growth across varying nitrate concentrations. The *narG* operon also includes two homologs of the nitrate/nitrite antiporter gene *narK*, as well as PA3871 (*nifM*) and *moaA1*, which encode molybdopterin cofactor synthesis enzymes, and *narHJI*, encoding additional subunits of the membrane-bound nitrate reductase (Palmer et al., 2007).

#### 4.1.2 Arginine fermentation regulatory genes

The *arc* operon encodes three key enzymes in the ADI pathway, *arcA* (arginine deiminase), *arcB* (catabolic ornithine carbamoyltransferase), and *arcC* (carbamate kinase), which is induced under oxygen-limited conditions (Vander Wauven et al., 1984). Subsequent studies revealed that the *arc* operon also includes the *arcD*, encoding a hydrophobic membrane-associated protein involved in the ADI process (Lüthi et al., 1990). Proteomic analyses of hypoxic *P. aeruginosa* biofilm regions reveal increased proteins linked to L-arginine and polyamine metabolism, with elevated ArcA and ArcB indicating active arginine-based energy production. Concurrently, the abundance of the cytosolic aminopeptidase PepA is approximately threefold higher in hypoxic compared to aerobic regions. PepA likely contributes to cellular protein degradation, recycling amino acids for stress responses such as pH homeostasis and energy generation (Pulukkody et al., 2021). Furthermore, the DNA-binding protein HupB is eight times more abundant in hypoxic zones relative to aerobic zones (Pulukkody et al., 2021). HupB, a small histone-like protein also known as heat-unstable (HU) protein, protects DNA from oxidative damage and facilitates adaptation to stress (Stojkova et al., 2019). In mammalian hosts, chronic *P. aeruginosa* infections are modulated by L-arginine metabolism and its derivative NO, with enhanced arginine catabolism observed at chronic wound sites (Debats et al., 2006). Microaerobic environments typical of chronic infection sites favor L-arginine fermentation, leading to NO deficiency, a hallmark of diminished host defense (Grasemann and Ratjen, 2012). Interestingly, L-arginine also supports phenazine production, and arginine metabolism remains largely unexplored (Ha et al., 2011).

#### 4.1.3 Pyruvate fermentation regulatory genes

A genome-wide analysis of anaerobic metabolism in *P. aeruginosa* identified several key pyruvate-metabolizing genes, including NADH-dependent lactate dehydrogenase (*ldhA*), phosphotransacetylase (*pta*), and acetate kinase (*ackA*) (Eschbach et al., 2004). The conversion of pyruvate to lactate and acetate relies on the intact *ldhA* and *ackA-pta* gene clusters, respectively (Eschbach et al., 2004). The anaerobic induction of the *ackA-pta* promoter is modulated by oxygen tension through the transcriptional regulators Anr and the integration host factor (IHF) (Eschbach et al., 2004). IHF, potentially encoded by the *anr* gene, is believed to contain a 4Fe-4S cluster that functions as an oxygen tension sensor (Galimand et al., 1991; Sawers, 1991; Green et al., 2001). The *ihfA* locus encodes a subunit of the DNA-bending IHF protein, which plays a role in transcriptional regulation (Delic-Attree et al., 1995, 1996). Additionally, the *gacS-ldhA* operon (PA0926 and PA0927) was identified, encoding the sensor kinase GacS of the GacA/GacS two-component regulatory system and a putative fermentative lactate dehydrogenase (LdhA), respectively (Eschbach et al., 2004). The *ackA-pta* locus (PA0835 and PA0836) likely encodes acetate kinase and Pta, respectively, while the *adhA* locus (PA5427) encodes a putative alcohol dehydrogenase (AdhA) (Eschbach et al., 2004). Under oxygen-limited conditions, *adhA* is induced to facilitate ethanol catabolism, contributing to anaerobic energy metabolism (Crocker et al., 2019).

### 4.2 Quorum sensing

*P. aeruginosa* utilizes quorum sensing (QS), a cell-density-dependent intercellular communication system, which plays a pivotal role in regulating bacterial virulence and biofilm formation (Lee and Zhang, 2015). Two principal QS signaling molecules, N-butyryl-L-homoserine lactone (C4-HSL) and N-(3-oxododecanoyl)-L-homoserine lactone (3O-C12-HSL), mediate QS through the transcriptional activator pairs *lasR*/*lasI* and *rhlR*/*rhlI*, respectively. Notably, the expression of *lasI* and *rhlI* is significantly upregulated under low oxygen conditions (Alvarez-Ortega and Harwood, 2007b). The QS system modulates genes involved in denitrification, thereby influencing bacterial growth and survival by regulating NO levels within the biofilm. Additionally, QS activates the transcription of the *pel* gene, partially through the Rhl system. The *pel* gene product synthesizes the glucose-rich extracellular polysaccharide matrix essential for *P. aeruginosa* biofilm formation (Friedman and Kolter, 2004; Sakuragi and Kolter, 2007).

In the CF airway environment, optimal expression of the *rhl* QS component benefits *P. aeruginosa* persistence. QS controls the expression of the *snr*-1 gene, which in turn regulates the denitrification rate (Hassett et al., 2002). Specifically, the *rhl* system acts as an anaerobic repressor of *snr*-1 transcription. Consequently, in the absence of RhlR, the reducing power provided by Snr-1 increases, thus enhancing NAR activity. However, dysregulation of denitrification genes in *rhlR* mutants leads to elevated transcription of *nar* and *nir* genes, causing accumulation of toxic NO and subsequent self-damage (Yoon et al., 2002). NO, a by-product of anaerobic respiration, accumulates in *rhlR* mutants despite a modest increase (2-fold) in NOR activity, which is insufficient to mitigate NO toxicity (Yoon et al., 2002). Thus, under anaerobic conditions, *P. aeruginosa* relies on the *rhl* QS system and NO reductase to regulate NO levels and sustain robust biofilm formation and survival (Yoon et al., 2002).

### 4.3 3,5-cyclic diguanylic acid (c-di-GMP)

Another crucial signaling molecule in *P. aeruginosa* is the second messenger 3,5-cyclic diguanylic acid (c-di-GMP), which facilitates bacterial adaptation to diverse environmental conditions (Ha and O’Toole, 2015; Römling et al., 2013). The c-di-GMP plays a central role in regulating biofilm formation and dispersion (Römling et al., 2013). Elevated intracellular c-di-GMP levels drive the transition from a planktonic to a biofilm lifestyle by repressing motility-related genes, including those regulated by FleQ, and activating genes involved in exopolysaccharide production and biofilm maturation (Baraquet and Harwood, 2013). Conversely, reduced c-di-GMP levels trigger biofilm dispersion by activating motility structures, including flagella and pili (Ha and O’Toole, 2015). The synthesis and degradation of c-di-GMP are catalyzed by diguanylate cyclases (DGCs) and phosphodiesterases (PDEs), respectively (Kalia et al., 2013). Although the interplay between QS and c-di-GMP signaling in *P. aeruginosa* is not fully elucidated, both pathways regulate virulence and biofilm dynamics, suggesting potential crosstalk. Current evidence indicates that the QS system can modulate intracellular c-di-GMP concentrations (Kim et al., 2018). Specifically, the Las-QS system may elevate c-di-GMP levels by stimulating DGC activity, whereas the Rhl-QS system might decrease c-di-GMP by inducing PDE activity. Furthermore, the tyrosine phosphatase TpbA inhibits the activity of the DGC TpbB via dephosphorylation, thereby reducing biofilm formation. Las-QS positively regulates TpbA expression, while Rhl-QS does not influence it (Ueda and Wood, 2009). Interestingly, TpbA also enhances *rhl* transcription, indicating that QS can negatively regulate c-di-GMP production in *P. aeruginosa*. However, the exact dynamics of c-di-GMP synthesis under varying QS states remain unclear. Moreover, NO has been reported to modulate DGC and PDE activities (Cutruzzolà and Frankenberg-Dinkel, 2016; Park et al., 2020). NO inhibits biofilm formation by enhancing PDE activity, leading to decreased intracellular c-di-GMP levels (Cutruzzolà and Frankenberg-Dinkel, 2016).

### 4.4 Transcription factors

TFs play a crucial role in regulating gene expression in response to environmental changes. Bacteria sense oxygen-limited conditions in their environment through Anr or Dnr, and regulate the expression of a series of genes to enable the bacteria to colonize and grow in oxygen-limited environments.

#### 4.4.1 Anr

Anr is a well-characterized global transcriptional regulator and a key activator of gene expression under hypoxic conditions. It governs a regulatory network of hypoxia-responsive genes and serves as a hallmark of anaerobic or microaerobic growth (Trunk et al., 2010). Under low oxygen tension, active Anr promotes *P. aeruginosa* biofilm formation and virulence, playing a crucial role in host colonization (Jackson et al., 2014; Hammond et al., 2015). Deletion of the *anr* gene results in defective biofilm development and often abolishes anaerobic growth, whereas elevated Anr activity enhances biofilm formation (Jackson et al., 2013). Nevertheless, the precise underlying mechanisms remain incompletely understood. Notably, Anr regulon genes show no significant transcriptional increase under hypoxia, consistent with the findings of Alvarez-Orgeta et al (Alvarez-Ortega and Harwood, 2007b), suggesting Anr regulation involves mechanisms beyond its own regulon transcription.

Anr is indispensable for activating energy metabolism pathways during hypoxia. It strongly induces denitrification and regulates the expression of other transcriptional regulators, including *dnr* and the *narXL* two-component system (Jackson et al., 2014). The *dnr* is essential for activating denitrification during anaerobic growth, controlling a subset of genes involved in nitrate respiration (Ferrara et al., 2021). The NarL response regulator modulates energy metabolism under hypoxic stress by promoting nitrate utilization and repressing less efficient energy-yielding pathways such as pyruvate and arginine fermentation (Schreiber et al., 2007; Benkert et al., 2008). Interestingly, while Anr enhances NarL production, it also independently promotes arginine fermentation by upregulating the arcDABC operon under anaerobic conditions (Filiatrault et al., 2006). Furthermore, Anr activates the *ackA-pta* operon responsible for pyruvate fermentation in response to low oxygen (Eschbach et al., 2004; Filiatrault et al., 2006).

Anr also upregulates genes encoding high-affinity cytochrome oxidases (*hemF* and *hemN*) and CupA fimbriae components (*oprG* and *cupA1-5*), facilitating respiratory adaptation during hypoxia and contributing to biofilm development and pathogenicity (Hammond et al., 2015). Additionally, Anr influences quorum sensing by regulating the small regulatory RNA PhrS (Sonnleitner et al., 2011), thereby modulating biofilm adaptability under low oxygen.

A well-studied virulence factor of *P. aeruginosa*, the hemolytic phospholipase C (PlcH), is tightly regulated by Anr. The catabolism of choline released by PlcH enhances Anr activity (Massimelli et al., 2005; Jackson et al., 2013, 2014). The plcH promoter contains a conserved Anr consensus binding sequence across all *P. aeruginosa* genomes (Galimand et al., 1991; Trunk et al., 2010). Anr represses plcH expression, maintaining PlcH protein at levels that facilitate effective host-pathogen interactions without compromising biofilm integrity (Jackson et al., 2014). Under oxygen-limited conditions, PlcH production may become disadvantageous, as excessive PlcH protein can compromise the structural integrity of the *P. aeruginosa* biofilm. Anr likely binds directly to the *plcH* promoter, which contains a conserved Anr consensus sequence across all *P. aeruginosa* genomes. Mutations in this conserved sequence result in increased *plcH* expression under hypoxia. Although Anr shares its consensus binding sequence with the secondary regulator Dnr, their activation mechanisms differ; notably, Dnr does not participate in *plcH* repression (Jackson et al., 2014). Anr is active when its 4Fe-4S iron-sulfur cluster remains intact, whereas Dnr is activated by the oxidation of its heme cofactor by nitric oxide, which induces a conformational change enabling DNA binding (Rodionov et al., 2005; Trunk et al., 2010).

The small RNA ErsA also plays an important role in anaerobic adaptation. It regulates bacterial-host interactions, including biofilm maturation, motility, and antibiotic resistance (Falcone et al., 2018; Zhang et al., 2017; Sonnleitner et al., 2020). ErsA is transcriptionally induced under oxygen-limited conditions (Ferrara et al., 2015) and transmits low-oxygen signals to the Anr regulon. It positively regulates Anr expression at the post-transcriptional level (Ferrara et al., 2021). Once ErsA surpasses a certain threshold, the RNA-binding protein Hfq acts synergistically with ErsA to activate Anr. This function of Hfq complements its own post-transcriptional regulation of Anr, indicating that ErsA-mediated activation of *anr* expression depends on Hfq (Ferrara et al., 2021). Hfq’s chaperone activity likely promotes the interaction between ErsA and *anr* mRNA, enhancing *anr* mRNA translation, possibly by improving access to its initiation site. This positive regulation by ErsA contributes to the stabilization of anr mRNA, consistent with the observed reduction in anr mRNA levels when ErsA is absent. Additionally, Hfq has been reported to stimulate anr expression via an unknown mechanism (Sonnleitner et al., 2011; Sonnleitner and Bläsi, 2014). Deletion of ErsA leads to reduced virulence of *P. aeruginosa* both *in vitro* and *in vivo*, markedly impaired biofilm formation and maturation (Ferrara et al., 2020), and severely compromised anaerobic growth through denitrification and arginine fermentation. The role of ErsA in biofilm regulation may also involve downregulation of the AlgC enzyme (Ferrara et al., 2015) and the activation of the AmrZ regulon (Falcone et al., 2018). Furthermore, ErsA directly negatively regulates *oprD* mRNA, affecting the envelope composition of *P. aeruginosa* (Zhang et al., 2017; Sonnleitner et al., 2020). These findings suggest that *P. aeruginosa’*s adaptation to the CF lung environment may increase its reliance on ErsA for regulating anaerobic metabolism.

#### 4.4.2 DNR

DNR is a critical transcriptional activator essential for initiating the denitrification process in *P. aeruginosa*. It was the first protein identified to restore anaerobic respiration and arginine substrate-level phosphorylation growth in *anr* mutants (Hassett et al., 2009). The expression and activity of Dnr are themselves regulated by Anr, positioning Dnr downstream in the oxygen-sensing regulatory cascade. Under hypoxic conditions, both Anr and Dnr coordinate to activate transcription of genes involved in denitrification and anaerobic respiration. Key operons under their control include *narKGHJIm*, encoding nitrate reductase; *nirSM-CFLGHJEN*, encoding nitrite reductase; and *ccoN_2_O_2_Q_2_P_2_*, encoding the *cbb_3–_* 2 cytochrome oxidase complex.

In the presence of nitrate under anaerobic conditions, expression from the *narK1* promoter is induced through the combined action of Anr, Dnr, and the nitrate-responsive two-component regulatory system NarXL (Schreiber et al., 2007). The DNA-bending protein integration host factor (IHF) is also crucial for optimal promoter activity. Moreover, Anr and NarXL induce *dnr* expression, amplifying the regulatory cascade (Schreiber et al., 2007). The cooperative function of NarXL and Dnr, regulated by ANR, is necessary for transcription of the nitrite reductase regulatory gene *nirQ* under anaerobic conditions (Schreiber et al., 2007; Hassett et al., 2009). Dnr belongs to the Crp-Fnr superfamily of transcriptional regulators and has been reported to activate expression of genes involved in the denitrification pathway, including *nir*, *nor*, and *nos* (Hasegawa et al., 1998; Arai et al., 2003). Transcriptional control of the *nar* locus occurs via the intergenic region between *narXL* and *narK1*. Both nitrate and nitrite induce *narK* expression, and a basal induction persists even during arginine fermentation (Schreiber et al., 2007). The *narK1* promoter activity is modulated by both Anr and Dnr, with Anr being indispensable for its baseline activation, while Dnr enhances promoter activity independently of Anr (Schreiber et al., 2007). Anr indirectly facilitates transcriptional activation of *nirS* by inducing *dnr* expression in this regulatory hierarchy. The transcription of NorC also requires Anr and Dnr in the presence of nitrous oxide but is not directly regulated by NarL (Arai et al., 1995).

Although the small RNA ErsA positively regulates Anr post-transcriptional (Ferrara et al., 2021), no direct regulation of Dnr by ErsA has been observed. Nevertheless, *dnr* transcript levels decrease in the absence of ErsA, likely due to diminished Anr expression. Additionally, under anaerobic conditions, expression of the *narXL* genes is also upregulated by both Anr and Dnr (Schreiber et al., 2007).

### 4.5 Two-component regulatory system

Two-component systems (TCSs) are widespread in prokaryotic genomes and constitute a fundamental regulatory network that enables bacteria to adapt, survive, and modulate pathogenicity in response to environmental changes. Functioning as molecular switches, these systems sense external stimuli and regulate gene expression accordingly, facilitating bacterial adaptation to diverse conditions. A canonical TCS consists of a membrane-bound sensor kinase (SK) and a cytoplasmic response regulator (RR), typically encoded adjacently in the genome (Linsky et al., 2020). Upon sensing environmental signals, the sensor domain of the SK undergoes conformational changes that are transmitted through the transmembrane region to its cytoplasmic histidine kinase domain. This triggers autophosphorylation of a conserved C-terminal histidine residue in trans (Mitrophanov and Groisman, 2008; Jacob-Dubuisson et al., 2018). The phosphoryl group is then transferred to a conserved aspartate residue on the RR, activating it. The activated RR modulates the transcription of downstream genes involved in diverse physiological processes, including bacterial virulence, pathogenesis, biofilm formation, cell division, and metabolite production (Tierney and Rather, 2019; Li et al., 2023).

In *P. aeruginosa*, the NarXL system is a nitrate-responsive two-component regulatory system (Krieger et al., 2002). NarX is the sensor kinase and NarL its response regulator; in the presence of nitrate, NarXL activates and cooperates with Dnr to upregulate *nar*, *nir*, and *nor* genes encoding nitrate, nitrite, and nitric oxide reductases (Hassett et al., 2009). Simultaneously, NarL represses the arginine fermentation pathway by inhibiting the arginine-dependent activation of the *arcDABC* operon mediated by the transcriptional activator ArgR. Specifically, NarL suppresses the expression of *arcA*, *arcB*, and *arcC* genes without affecting the oxygen tension-dependent activation driven by Anr (Benkert et al., 2008). Under conditions where both nitrate and arginine are present, NarL binding likely interferes with ArgR’s interaction at overlapping DNA binding sites, thereby preventing ArgR-mediated induction of *arcDABC* transcription (Benkert et al., 2008).

### 4.6 Other factors

In addition to above factors, studies have identified other specialized genes that promote *P. aeruginosa* growth and biofilm formation. OmpW is an eight-helix β-barrel outer membrane porin that facilitates the uptake of small hydrophobic molecules (Catel-Ferreira et al., 2016). It has been demonstrated that OmpW participates in bacterial adaptation to various environmental stresses (Brambilla et al., 2014). In *P. aeruginosa*, OmpW expression is upregulated under hypoxic or anaerobic conditions (McPhee et al., 2009). However, OmpW expression may be downregulated when iron levels are low, as OmpW is implicated in iron uptake (Lin et al., 2008). Concurrently, under hypoxia, the global regulator Lrp is upregulated (Gil-Marqués et al., 2022). The *ompW* promoter contains an Lrp-binding site, through which Lrp negatively regulates *ompW* expression (Gil-Marqués et al., 2022).

Another crucial outer membrane protein, OprF, functions as a cytokine and plays a vital role in regulating anaerobic metabolism in *P. aeruginosa.* It is essential for the optimal survival of anaerobic biofilms (Hassett et al., 2002). The absence of OprF results in severely impaired bacterial growth due to the loss of nitrite reductase activity and defects in anaerobic respiration. Notably, OprF is detectable exclusively in anaerobic biofilms (Beg et al., 2023), and only CF patients with chronic infections possess antibodies against OprF. Bacteria lacking *oprF* exhibit diminished anaerobic biofilm formation, partly attributable to the lack of NIR activity. Two hypotheses have been proposed regarding OprF’s precise role in anaerobic growth. First, OprF may serve as a porin facilitating nitrate or nitrite transport into the cell and potentially interact directly with NIR to stabilize its enzymatic activity. Second, the absence of OprF may compromise peptidoglycan stability, as OprF has been shown to interact with this essential cell wall component (Rawling et al., 1998). Reduced peptidoglycan integrity renders cells more fragile and susceptible to environmental stress.

## 5 Clinical significance and future directions

Biofilm formation serves as a crucial protective strategy, enabling pathogenic bacteria to resist various environmental stresses (Ciofu and Tolker-Nielsen, 2019). The hypoxic microenvironment within biofilms facilitates bacterial adaptation through reduced metabolic activity and heightened antibiotic tolerance (Penesyan et al., 2015; Stokes et al., 2019), which contributes to persistent chronic infections. Traditional antibacterial treatments have largely targeted the eradication of pathogens but often overlook the modulation of the infected microenvironment, resulting in issues such as antibiotic resistance and incomplete bacterial clearance (Hou et al., 2021; Zhao et al., 2023b). Recently, strategies aimed at reversing the hypoxic microenvironment have emerged as a promising research focus for combating biofilm-associated infections.

Reversal of hypoxia triggers a cascade of beneficial effects, including the reactivation of suppressed immune responses, promotion of osteogenesis and angiogenesis, induction of cuproptosis-like bacterial death, and stimulation of dendritic cells and macrophages to enhance antibacterial activity via chemotaxis and phagocytosis (Luo et al., 2024). Photodynamic therapy (PDT) is a notable antibacterial approach capable of effectively killing bacteria and preventing multidrug resistance; however, its efficacy is markedly compromised under hypoxic conditions. Enhancing oxygen delivery to treatment sites and alleviating hypoxia significantly improves PDT efficacy, providing a promising avenue for biofilm eradication (Xiu et al., 2020; Bai et al., 2021; Wu et al., 2022).

Recent advancements include the development of porphyrinic metal-organic framework (MOF)-based metalloantibiotics that catalyze endogenous hydrogen peroxide (H_2_O_2_) decomposition to generate oxygen. The resulting oxygen enhances oxygen-dependent sonodynamic therapy (SDT), which disrupts bacterial homeostasis—affecting cell membrane integrity and quorum sensing systems—thereby promoting bacterial killing (Su et al., 2024). Concurrently, sustained oxygen production supports fibroblast survival and migration, stimulates angiogenic growth factors, promotes angiogenesis, and increases secretion of anti-inflammatory cytokines (Su et al., 2024). Nitric oxide (NO) exhibits dual effects depending on its concentration: high levels possess bactericidal activity, whereas low levels induce biofilm dispersion and sensitize bacteria to antibiotics (Cai et al., 2021). In *P. aeruginosa* models, low-dose NO, which is non-lethal, acts as a signaling molecule triggering biofilm dispersal in ex vivo cystic fibrosis (CF) sputum, reducing bacterial tolerance to tobramycin alone or combined with ceftazidime. This highlights NO’s potential as an adjunct therapy for managing *P. aeruginosa* biofilm infections in CF patients (Howlin et al., 2017). Consequently, NO-based treatments represent a promising approach against antibiotic-resistant bacteria and biofilm-associated infections. Overall, alleviating biofilm hypoxia holds significant potential for enhancing treatment efficacy and overcoming chronic biofilm-related infections.

## 6 Conclusions

Biofilm cells exhibit an oxygen gradient that typically reduces their sensitivity to antibiotics, making complete eradication challenging. The adaptation of bacterial biofilms to hypoxic conditions is considered a crucial factor for their prolonged latent persistence in the human body. This hypoxic microenvironment triggers a series of complex bacterial responses. Current studies suggest that under oxygen-limited conditions, *P. aeruginosa* shifts to anaerobic metabolism, utilizing nitrate for denitrification to support growth. In the absence of nitrate or nitrite, survival is maintained through arginine or pyruvate fermentation pathways. Additionally, *P. aeruginosa* biofilms produce phenazine compounds to sustain redox balance within the biofilm matrix. Adaptation to oxygen limitation also involves the regulation of virulence gene expression and QS systems. These processes are coordinately controlled by transcriptional regulators such as Anr, Dnr, NarXL, and other specialized genes, collectively promoting bacterial survival and biofilm formation. A deeper understanding of the adaptive mechanisms employed by bacterial biofilms under oxygen-limited conditions can provide new directions for future treatments of biofilm-associated infections, including strategies targeting QS or c-di-GMP pathways, modulation of Anr or Dnr regulators, and nitric oxide-based therapies.
